# A Last Word

**Published:** 1899-04

**Authors:** E. K. Wedelstaedt

**Affiliations:** St. Paul.


THE
AMERICAN JOURNAL
—OF—
DENTAL SCIENCE.

VOL. XXXII. BALTIMORE, APRIL, 1899. NO. 12.

### Selections.

ARTICLE I.

### A LAST WORD.

E. K. WEDELSTAEDT, D. D. S., ST. PAUL.

In the fewest words possible, I desire to close the discussion of Chapter XII, American Text Book. There are two things that it seems necessary for me to state. First—I do use cement, oxy phosphate (and oxy-chloride also), but I do not use it in every cavity in which I am going to make a metal filling. I do cap pulps with soft cement for many children under fourteen years of age with the feeling that the pulp may live, but with the belief that it will die. Secondly—I do not know Dr. Clapp, have only the most friendly feeling for him, and I am not criticizing, as Dr. Ottolengui says, "the man, but his methods" and the principles which he has seen fit to publish. Dr. Clapp advances these ideas, viv.: That we should first fill those cavities in which metal fillings are to be placed, with soft cement, filling the cavity two-thirds full, and then into the soft cement crowd pieces of the filling material (gold or amalgam), either of which we may be about to use to fill the tooth permanently. After the cement has hardened, trim away from the margins the cement that has been forced from the cavity by placing the first pieces of our metal filling into the cement. The margins must be free from cement. Then continue to pack our filling material against that already anchored in the soft cement. Sole reliance is placed on the anchorage of the first pieces in the cement. Not any other mechanical or supplementary anchorage is made. This is the sum and substance of what I have been criticizing. I wish my readers to bear this in mind. All that is necessary to say in condemnation of principles and methods of this kind has been said in my former articles. I would not have repeated this much, but I wish my position to be clearly understood.

Exception has been taken by some to the published results of my experiments with Weston's insoluble cement. This should have been explained. Some time before anything was published in regard to this subject. I wrote Dr. Clapp and asked what filling material he used to gain the ideas he saw fit to publish. He wrote and told me a good combination filling of amalgam and cement could be made by using Weston's cement and Fellowship alloy. The Fellowship alloy I was using at the time set in ninety seconds after it was amalgamated, and I made up my mind that Dr. Clapp either had a different kind of Fellowship from that I was in the habit of using, or else he was a good deal of a joker. (I have learned within a week that there are two kinds of Fellowship, slow and rapid setting). This is why I made some experiments with Weston's cement, and the above also tells why I did not make combination amalgam and cement fillings using Fellowship alloy.

And as my experiments with combination amalgam and Weston's cement fillings have closed, I do not desire at this time to open them again. Since my last paper was written I have spent somewhat over six weeks in my laboratory investigating cements. I have made about three thousand experimental fillings, and I think about one thousand experiments, more or less. Twenty five or more different oxy-phosphates have been tested. Also a few oxy-chlorides. Green teeth have had carefully prepared cavities made in them and then filled with Weston's cement. After the cement had set, these were placed in water that had a trace of aniline in it. The water in some cases was merely at room temperature, and in other at 98° F. These have been allowed to stay in the bath for twenty-four, forty eight and seventy-two hours, with what result? Only to discover that traces of aniline could be found in every portion of the cement. Very often the aniline could be traced clear around the axial wall. In one case where a cavity in a green tooth had been filled with Weston's cement, after the cement had fully set, it was placed in a glass of water at room temperature, and in just thirty-six hours there was not a trace of the cement left in the cavity. It had been dissolved out.

Dr. Ridout made and fitted three gold crowns to the roots of three green teeth. Dr. Ridout is a man of great skill in this particular line of work, and the crowns were as perfect as human ingenuity could make them. They were seamless, and Dr. Ridout cemented them to the roots, using We ton's cement. These were placed in water at 98° F., for twenty-four, forty-eight and seventy-two hours, with what result? Only to find on cutting the crowns from the roots that the longer they remained in the bath the greater was the penetration of moisture.

I made a series of twelve fillings from one mix and mass of Weston's cement. Six of the fillings were tested dry, and six were tested after being in a bath for different periods of time. A comparative test in regard to their ability to withstand pounds pressure in the dynamometer was made; also a test to note the penetration of moisture. Every filling of Weston's cement that was examined after being in the bath showed traces of aniline from circumference to center. And when it came to comparing the amount of stress the wet and dry fillings carried—well, the least said the better.

Green teeth were then taken, molars, cuspids and incisors. These had carefully prepared cavities cut in them. The margins in every case were most carefully planed with sharp chisels. These were filled with combination fillings, a la Clapp, Weston's cement, and different makes of alloy. After the fillings had been made, they were placed in water at 98° F., for twenty-four, forty-eight and seventy-two hours. (There was always a series of three fillings made). After they were removed from the moisture, they were either broken in a vise or the filling was pushed out by placing the tooth in the dynamometer and exerting stress on it. And now what were the results? I discovered that moisture penetrated the cement of all but one, and in this one the moisture had just reached the cement. This tooth was in the bath but twenty-four hours, was an upper molar, and the cavity had a broad, flat seat at the gingiva. I also discovered that the thinner the veneer of amalgam the greater the pentration of the moisture into the cement. I discovered too, that these same thin veneer fillings of amalgam would frequently carry five pounds of stress before the amalgam would break from the cement. This took place with three fillings made in incisors. Three proximal combination fillings of amalgam and cement in cuspids carried twenty and twenty-five pounds respectively before being pushed out. A lateral incisor with as perfect a filling as I could make by this method carried thirty pounds before the anchorage with the cement was broken. Three large proximal cavites in molars were prepared, and combination fillings of amalgam and cement made in them. The cavities in these were seven, eight and nine millimeters respectively, linguo-buccogingivally by from three to five millimeters deep from the pulp to the margin (disto-pulpally). These carried one hundred and fifty, one hundred and ninety and two hundred pounds. The fillings did not break from the cement, for the reason that the amalgam was pushed against the axial wall in each case. But they did break where the amalgam had been packed to the amalgam that had originally been anchored in the cement. This is just where the greatest strength is required, but at the exact moment it was needed, it failed. Dr. Clapp may be like my friend Dr. Flagg, of Philadelphia, who said, "No man has power enough in the muscles of his jaw to bite forty pounds with his teeth." I believe Dr. Jones, of New Haven, quickly demonstrated to Dr. Flagg's satisfaction that he could press on the gnathodynamometer, not forty, but two hundred and fifty pounds with his molars, and then not make very much of an effort. So do not be deluded; men can and have repeatedly closed my machine (three hundred and forty pounds) with their molars.

I am glad I went into this controversy, for the dental profession is going to have better cements. The makers of the cements have awakened to the realization that every man is liable to test the cement he is using, by making a filling and dropping it into a bottle of ink to note if it is penetrated, and if so they do not wish to use it. And there is little need of its being penetrated. There are water or motsture-proof cements to be had, and these we wish and will have.

I am unwilling to discuss again the partly prepared and filled cavity (combination filling, gold and cement), sent by Dr. Clapp to Dr. Ottolengui, criticized by him and then sent to me. It is sufficient for me to know that every fundamental principle governing the packing of gold was violated in packing the gold this cavity contains. It is not my fault that Dr. Ottolengui did not notice this nor the condition of the cement. In regard to the "proper application of the method," I desire to say there is no such thing as "properly applying" this method, if the rules laid down in the Text Book are followed. Dr. Clapp tells, as I have said before, his own story in writing and illustrations. They are all of the past, have long since been condemned and cannot bear a scientific investigation. So the least said the better.

I give the readers the results, for I have not the time to enter into particulars in regard to the work I have been doing. I have spent too much time on this work as it is. It has cost me a fortune to obtain these results, and so I must begin to attend to my long neglected business. I ask of that portion of the dental profession who are making combination fillings of gold and cement, or thin veneer amalgam fillings, anchored in soft cement to fill a cavity in a freshly extracted or green tooth by this method, and then subject to a bath of ordinary water that has a trace of aniline in it, at room temperature, for twenty-four or forty-eight hours. At the end of this time, remove tooth, wash it in ordinary water and dry in muslin. Now place the tooth in a vise and crack it. Remove the filling and examine the cement, using a one inch power in the microscope. It will tell its own story. The surprises in store for those making this experiment will be sufficient for each and every one making it. Just as surely as there is penetration of the aniline into the cement, just that certain may the experimenter be that the fluids of the mouth will also penetrate the cement. We now know *why* the cement under so many of our gold and amalgam fillings stink so badly, why so many cement fillings stink, and why the cement in so many gold crowns is in such bad condition. The why and proof we have. But this will all shortly be different, for the makers of some of our cements have already improved their wares, and others will also do so.—*Items of Interest.*

